# Laparoscopic Heated Intraperitoneal Chemotherapy in the Treatment of Carcinomatosis of Gastric Adenocarcinoma Origin

**DOI:** 10.3390/jcm10204757

**Published:** 2021-10-17

**Authors:** Michael G. White, Brian D. Badgwell

**Affiliations:** Department of Surgical Oncology, The University of Texas MD Anderson Cancer Center, Houston, TX 77030, USA; mgwhite@mdanderson.org

**Keywords:** carcinomatosis, HIPEC, gastric adenocarcinoma, regional therapies

## Abstract

The use of heated intraperitoneal chemotherapy (HIPEC) in conjunction with cytoreductive surgery has been gaining increasing traction in treating gastric adenocarcinoma with metastasis to the peritoneum in recent years. The addition of laparoscopic HIPEC (LS-HIPEC) to these treatment algorithms has increased the flexibility and adaptability of HIPEC integrating into treatment sequencing, allowing for iterative protocols of LS-HIPEC prior to cytoreduction as neoadjuvant treatment, as well as in the palliation of patients with unresectable disease and uncontrolled ascites. As the use of HIPEC in gastric adenocarcinoma continues to be refined, LS-HIPEC algorithms should continue to be considered and utilized both in curative treatment algorithms as well as in patients in the palliative setting. Given that LS-HIPEC remains a relatively nascent treatment modality, we advocate for its use in the setting of a clinical trial when feasible.

The use of heated intraperitoneal chemotherapy (HIPEC) in managing carcinomatosis of gastric origin was first described by Fujimoto and colleagues in 1988 [1]. Since that time, these treatment algorithms have undergone significant study and refinement through a variety of observational studies and clinical trials. Nevertheless, the peritoneal spread of gastric adenocarcinoma is common, accounting for 49% of recurrences [2], and is associated with an overall poor prognosis despite modern systemic chemotherapy regimens with patients with M1 peritoneal disease surviving a median of 15 months [3]. Today these studies have resulted in improved patient selection, continued optimization of perfusate, the addition of minimally invasive techniques, and iterative neoadjuvant laparoscopic HIPEC (LS-HIPEC) protocols [4,5]. While primarily performed as a life-prolonging procedure, the low morbidity of LS-HIPEC has also allowed for its expanded use as a palliative procedure to control patients’ symptomatic malignant ascites. In this monograph, we review the role of LS-HIPEC in the treatment of gastric cancer, including converting patients’ disease from unresectable to resectable and palliating malignant ascites for patients receiving definitive systemic therapy. Finally, we discuss the early experience of pressurized intraperitoneal aerosol chemotherapy (PIPAC) as a recent additional tool in LS-HIPEC regimens [6].

As treatment algorithms defining the utility of HIPEC in the management of gastric cancer with peritoneal metastases began to become standardized in the 1990s, randomized control trials of cytoreductive surgery (CRS) for gastric cancer with and without HIPEC became feasible. The first of these randomized trials was completed by Yang et al., randomizing 68 patients to compare CRS (with gastrectomy) versus CRS + HIPEC with cisplatin and mitomycin C for 60–90 min via the coliseum technique. The addition of HIPEC demonstrated a statistically significant improvement in disease-specific survival from 6.5 to 11 months on uni- and multivariate analysis [7]. Moreover, this provided early subgroup analysis data demonstrating improved survival in those patients able to achieve an R-0 or R-1 resection. This set the stage for the fundamental tenets of CRS and HIPEC in patients with isolated peritoneal gastric adenocarcinoma. Notably, HIPEC had the potential to improve survival, but this improvement required an R-0 or R-1 resection.

Reviews of non-randomized trials studying CRS and CRS HIPEC in the peritoneal spread of gastric cancer have demonstrated several CRS HIPEC algorithms that have reported variable median survivals ranging from 9 to 20 months [5,8,9,10,11,12,13]. Most recently, a retrospective case-control study (CYTO-CHIP) studied 277 patients in 19 French centers. This again demonstrated significantly improved overall survival in the CRS + HIPEC group (21.2 months) compared to those receiving CRS alone (14.7 months). Intriguingly the impact of the addition of HIPEC played a particularly strong role in decreasing rates of peritoneal recurrence in those patients with isolated peritoneal disease or peritoneal and extraperitoneal disease [14]. Meanwhile, treatment with HIPEC was most efficacious in the setting of poorly cohesive (PCC) gastric cancers with a median survival for PCC gastric cancer undergoing CRS + HIPEC of 34.5 months versus 14.3 months in the PCC CRS alone group [15].

While randomized trials of CRS with or without HIPEC ask an important and nuanced question, they do not address the more fundamental question of the benefit of operative intervention in these metastatic patients versus standard of care chemotherapy. This is a particularly difficult question to answer given the rarity of this disease and the rate at which systemic therapy options are added or refined. The GYMSSA trial attempted to answer this question, although they failed to fully accrue their target number of patients. Those that were accrued had metastatic gastric cancer (peritoneal or otherwise) treated with FOLFOXIRI and were compared to patients with peritoneal disease undergoing CRS and HIPEC. Although 4 of the 11 patients in the CRS/HIPEC group lived beyond 4 years, this came at the expense of 11% perioperative mortality. Along failing to meet accrual, this trial had a significant limitation in that many patients had received multiple lines of pretreatment prior to enrollment, and patients with liver or lung metastases were included in the systemic therapy arm as well [16].

Building on these experiences of open CRS HIPEC, the safety of iterative neoadjuvant LS-HIPEC in a western population was demonstrated in a phase II trial at our institution for patients with low-volume disease or positive cytology at the time of diagnosis. In this trial, patients with gastric adenocarcinoma and positive washings or low-volume peritoneal disease were enrolled and offered iterative neoadjuvant LS-HIPEC. They then underwent laparoscopic exploration, washings, and biopsy followed by perfusion with 30 mg mitomycin C and 200 mg cisplatin. This was repeated (up to 5 times) until patients cleared their positive cytology and/or biopsies and proceeded to resection, or their disease progressed. In cases of progression, patients proceeded to further systemic chemotherapy or another clinical trial [17]. This work showed iterative LS-HIPEC was safe and converted patients’ disease from unresectable to resectable upon evaluation at the time of subsequent LS-HIPEC in 5/19 (26%) cases [5]. Since that time, an additional phase I trial of a 3-drug regimen adding paclitaxel to mitomycin and cisplatin demonstrated this three-drug regimen to be safe. Expanded patient cohorts have allowed for the retrospective identification of prognostic factors, such as the presence of preoperative ascites or a high PCI score that are negatively associated with conversion to resectable disease in patients undergoing neoadjuvant LS-HIPEC protocols [18,19]. As such, our practice has used these and other studies to optimize contemporary treatment algorithms for our patients. Our current treatment algorithm is summarized in Figure 1.

In optimizing patient selection, it was noted that few patients with ascites at the time of initial laparoscopy were able to clear their disease and proceed to resection [19]. Currently, we advise patients with pretreatment ascites against enrollment in these protocols in the absence of extenuating circumstances and have opted to continue the use of two-drug oxaliplatin and mitomycin given observed equivalency with the three-drug regimen [19]. The safety of cytoreduction for gastric cancer and the use of LS-HIPEC in these patient populations has been demonstrated across countries, institutions, and a variety of treatment algorithms have been proposed.

Until recently, life-prolonging algorithms have been the primary focus of study in LS-HIPEC. At the same time, increasing interest in quality of life and symptom control has been considered in patients who did not qualify for or were not interested in these trials. For those patients with peritoneal spread of their gastric cancer, a common manifestation is symptomatic ascites. In order to palliate symptoms resulting from these ascites, several groups have performed laparoscopic peritoneal perfusion for patients with debilitating malignant ascites. The minimally invasive nature of this procedure offers minimal interruptions in systemic treatment regimens and minimizes medical risk in patients that are typically medically frail. In these cases, patients with unresectable disease and ascites undergoing definitive systemic treatment can undergo LS-HIPEC to decrease their ascitic burden and minimize the need for paracenteses. Although no randomized trials exist to study the use of LS-HIPEC in the treatment of malignant ascites, it has been favorably reported in several series, offering an option to palliate this difficult problem [20,21,22]. These studies demonstrate that a significant reduction in ascites can be achieved in 91–94% of patients in reported series [22,23]. While rates of significant complications (Grade 3 or 4) are low, even in these medically frail patients, ranging from 2 to 12% [22,24]. Here we have seen clear evidence of symptomatic relief in case series of patients whose treatment options are extremely limited and who are often highly symptomatic. Given the limited options for these patients, consideration should be given to this treatment modality at high volume centers familiar with LS-HIPEC after an in-depth discussion of the associated risks and benefits.

Recent years have seen further innovation in the use of pressurized intra-peritoneal aerosol chemotherapy (PIPAC) to treat unresectable gastric cancer. This treatment modality works by delivering pressurized and aerosolized chemotherapy as it travels through a laparoscopic port to maximize penetrance and delivery to the tissues. This route is thought to minimize adverse effects and is performed as a life-prolonging maneuver in patients with unresectable disease. These studies are currently performed in the setting of clinical trials in phase I [25,26], phase II [27,28,29,30], and early phase III [31] settings. These trials offer the potential for life prolongation while potentially improving quality of life over standard systemic chemotherapy options [32,33]. To date, data exclusively studying PIPAC in gastric cancer is limited, with reported radiologic response rates of 40% [30] and histopathologic responses of 79% [34]. Efficacy is difficult to interpret in small cohorts defining safety. Ultimately, further work is needed to define the role of this treatment modality in gastric cancer in the peritoneal metastatic setting, as well as define its utility in potentially curative treatment algorithms as neoadjuvant or adjuvant therapy.

While treatment pathways for patients with localized gastric cancer are clear and offer a potential for cure, those patients with peritoneal disease offer a more challenging gambit. In patients with low-volume disease with the potential to convert to a candidate for resection with LS-HIPEC, various algorithms have been defined as safe but have not been well studied in contrast to definitive systemic therapies with or without radiotherapy. We currently advocated for an iterative neoadjuvant two-drug treatment regimen in patients with good performance status who we expect will complete this regimen and would tolerate a potential eventual gastrectomy. To date, however, the utility of LS-HIPEC in prolonging survival in gastric cancer remains unknown. Retrospective comparisons at our institution have failed to demonstrate a survival benefit when comparing iterative LS-HIPEC to the standard of care chemotherapy. Experiences at other institutions and a meta-analysis of randomized clinical trials, however, have shown a marked and significant improvement [35,36,37]. Despite these contradictory findings, we and others observe a minority of patients who have an excellent and occasionally durable response. We, therefore, feel that further rigorous study is needed through prospective clinical trials such as the ongoing DRAGON II, in which randomized patients with locally advanced (T4) gastric cancer receive either an R0 resection and D2 lymphadenectomy followed by eight cycles of SOX (Doxorubicin, Adriamycin, and oxaliplatin with S-1) or neoadjuvant LS-HIPEC with Paclitaxel followed by three cycles SOX and R0 resection with D2 lymphadenectomy [38]. PERISCOPE trials randomizing patients with T3-T4 gastric cancer with limited peritoneal disease or positive cytology were randomized to palliative systemic chemotherapy or CRS + HIPEC after 3–4 cycles of systemic chemotherapy [39]. Additionally, we must prioritize translational research to better define disease biology and inform patient selection and the development of new agents and treatment modalities in the management of an aggressive malignancy such as gastric cancer.

## Figures and Tables

**Figure 1:**
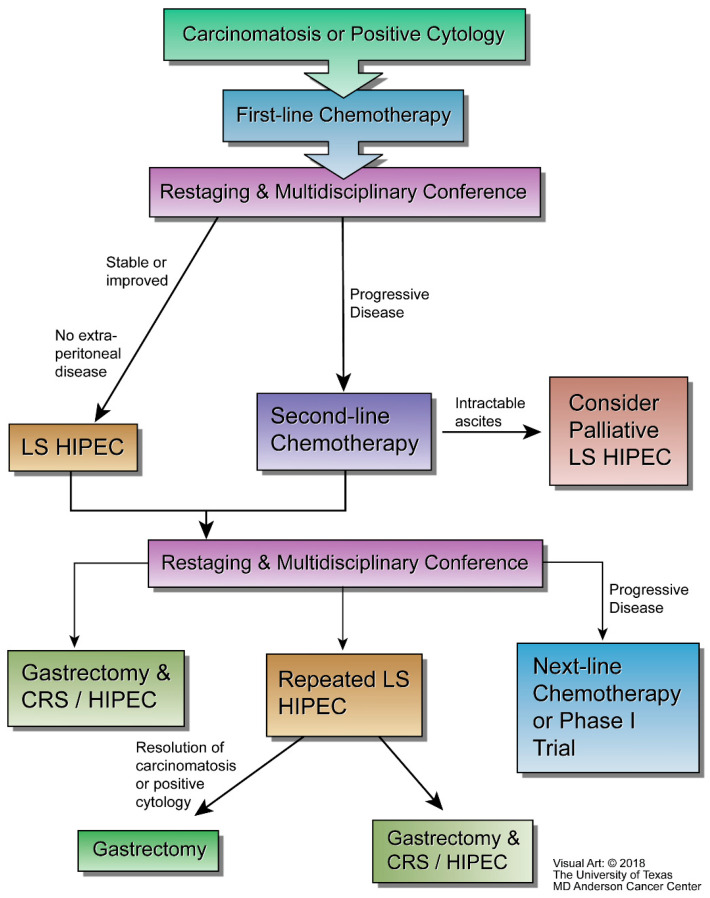
Flow chart of management of gastric cancer with carcinomatosis or positive cytology at MD Anderson Cancer Center.

## Data Availability

No new data were created or analyzed in this study. Data sharing is not applicable to this article.
